# Sex- and age-specific associations of serum essential elements with diabetes among the Chinese adults: a community-based cross-sectional study

**DOI:** 10.1186/s12986-024-00801-3

**Published:** 2024-07-09

**Authors:** Dongmei Wang, Hong Ye, Siyang Liu, Hualin Duan, Qintao Ma, Nanfang Yao, Zihao Gui, Genfeng Yu, Lan Liu, Heng Wan, Jie Shen

**Affiliations:** 1 Institute and Department of Endocrinology and Metabolism, Shunde Hospital, Southern Medical University (The First People’s Hospital of Shunde), No.1 of Jiazi Road, Lunjiao, Shunde District, Foshan City, 528308 Guangdong Province China; 2 School of Nursing, Southern Medical University, Guangzhou, Guangdong Province China

**Keywords:** Sex- and age-specific association, Copper, Magnesium, Essential elements, Diabetes

## Abstract

**Background:**

Although several studies have found the relationship between essential elements and diabetes, the studies about the association of essential elements with diabetes diagnosed according to an oral glucose tolerance test (OGTT) and glycated hemoglobin (HbA1c) in a sex- and age-specific manner were limited. To investigate the linear and nonlinear relationship of five essential elements including iron (Fe), copper (Cu), Zinc (Zn), magnesium (Mg), and calcium (Ca) with diabetes, fasting plasma glucose (FPG), 2-h postprandial plasma glucose (PPG), and HbA1c and to evaluate the sex- and age-specific heterogeneities in these relationships.

**Methods:**

A total of 8392 community-dwelling adults were recruited to complete a questionnaire and undergo checkups of anthropometric parameters and serum levels of five metals (Fe, Cu, Zn, Mg, and Ca). The multivariable logistic and linear regression, the restricted cubic spline (RCS) analysis, and subgroup analysis were applied to find the associations between the essential elements and the prevalence of diabetes as well as FPG, PPG, and HbA1c.

**Results:**

In the multivariable logistic regression and multivariable linear regression, serum Cu was positively associated with FPG, PPG, and HbA1c while serum Mg was significantly inversely correlated with FPG, PPG, HbA1c, and diabetes (all *P* < 0.001). In the RCS analysis, the non-linear relationship of Cu and diabetes (*P* < 0.001) was found. In the subgroup analysis, stronger positive associations of Cu with diabetes (*P* for interaction = 0.027) and PPG (*P* for interaction = 0.002) were found in younger women.

**Conclusions:**

These findings may lead to more appropriate approaches to essential elements supplementation in people with diabetes of different ages and sexes. However, more prospective cohort and experimental studies are needed to probe the possible mechanism of sex- and age-specific associations between serum essential elements and diabetes.

**Supplementary Information:**

The online version contains supplementary material available at 10.1186/s12986-024-00801-3.

## Introduction

Diabetes is a metabolic disorder characterized by hyperglycemia, insulin resistance, and relative insulin deficiency, resulting from the interaction of genetic and environmental factors [1]. In 2017, approximately 451 million people worldwide had diabetes, and by 2045, that number is expected to rise to 693 million [2]. Even worse, 27% of diabetic adults had cardiovascular disease, and half had diabetic microvascular complications, including diabetic kidney disease, diabetic retinopathy, and diabetic neuropathy [3]. As a result of its high prevalence, disability, and mortality, the disease has become a critical health concern worldwide [4]. Fasting plasma glucose (FPG) and 2-h postprandial plasma glucose (PPG) are of great clinical value in distinguishing diabetes from prediabetes [5].

The balance of essential elements was crucial to endocrine function [6]. Our previous studies found that blood magnesium (Mg) was positively associated with serum uric acid, dyslipidemia, and thyroid nodules [7–9]. What’s more, growing evidence has suggested that the imbalance of essential elements seems to be related to the onset and progression of diabetes [10]. A prospective cohort study included 5044 subjects from 15 provinces of China, had suggested the increasing risk of diabetes and insulin resistance when serum Mg decreases [11]. Also, a current study demonstrated that plasma Mg, iron (Fe), and copper (Cu) were closely related to FPG [12]. In addition, the positive correlation between plasma Cu and glycated hemoglobin (HbA1c) was explored in Chinese adults in a cross-sectional study [13]. Pittas, Anastassios G. et al. [14] reported that calcium (Ca) intake can reduce the risk of diabetes potentially. Nevertheless, another study indicated a positive correlation between serum Ca and the risk of diabetes [15]. What’s more, several studies have investigated that the heavy metals were sex-specific correlated with glucose levels, kidney function, and cancer mortality in US adults [16, 17]. A previous study in China also showed that the relationship between plasma Fe and FPG was negative in men but not in women [12]. Compared with FPG, more patients with diabetes and prediabetes were diagnosed by PPG [18]. However, the association of essential elements with PPG remains understudied, not to mention the studies with diabetes, FPG, PPG, and HbA1c simultaneously.

What’s more, multivitamin-mineral supplements, including essential elements, are increasingly being used for disease prevention and health care in recent years [19], particularly in the prevention and management of diabetes [20]. Studies have shown that 51% of US adults with diabetes self-report using any mineral supplement [20]. However, there is no clear evidence to suggest that dietary supplements can improve the outcome of diabetes [21], and research on whether there are sex-differences in the relationship between essential elements and diabetes is still limited.

Thus, in the current study, we selected five essential elements including Fe, Cu, Zinc (Zn), Mg, and Ca, which are frequently detected clinically, to explore their relationship with the prevalence of diabetes as well as different blood glucose status including FPG, PPG and HbA1c. Moreover, we investigated the non-linear relationship between essential elements and the prevalence of diabetes. Furthermore, sex- and age-specific heterogeneities in the associations were also evaluated.

## Methods

### Study design and population enrollment

We recruited volunteers in 2021 from Shunde District, Foshan, China, using stratified random sampling. Inclusion criteria included being older than 18 years old, not pregnant and living in Shunde for at least half a year. Of 13,535 potential volunteers, we excluded those who did not provide blood samples (*n* = 3412), those who had taken mineral supplements in the previous three months and had missing data on serum essential elements (*n* = 938), those with missing PPG values, HbA1c values, and fasting insulin values (*n* = 696), and those taking antidiabetic medication (*n* = 97). Finally, a total of 8392 participants were included in this study (Supplementary Fig. 1).

The Ethics Committee of Shunde Hospital of Southern Medical University approved the study protocol (20211103) according to the ethical guidelines of the 1975 Declaration of Helsinki. Informed and written consent was obtained from all participants before enrollment. The study was registered at www.chictr.org.cn (ChiCTR2100054130) as a cross-sectional investigation.

### Measurements

The sociodemographic characteristics, lifestyle characteristics, and medications of the participants were collected using a standard questionnaire administered by trained study personnel. Anthropometric parameters including body weight and height were measured according to a standard protocol. Body mass index (BMI) was calculated as weight in kilograms divided by height in meters squared (kg/m^2^). Blood pressure was measured by an automated electronic device (HEM-752 FUZZY, Omron, China) on the nondominant arm twice with a 10-minute interval following a 5-minute rest [22]. The average systolic and diastolic blood pressure of the two readings was calculated [1].

The fasting blood samples were collected from all participants from 08:00 to 10:00 after an overnight fast of at least 10 h. The whole blood samples were collected in vacuum tubes containing heparin sodium and used for the measurements of the essential element levels including by inductively coupled plasma mass spectrometer (ICAP-RQ, Thermofisher Scientific, USA).

The blood samples for the FPG and PPG levels after carrying out an oral 75 g glucose tolerance test were collected into vacuum tubes with the anticoagulant sodium fluoride. Among people with self-reported diabetes, only FPG and HbA1c were measured. HbA1c was assessed by high-performance liquid chromatography (HLC-723G8, TOSOH, Japan). Plasma glucose levels and serum lipid profiles including total cholesterol (TC), triglyceride (TG), high-density lipoprotein (HDL), and low-density lipoprotein (LDL) were conducted by BS800 (Mindray, Shenzhen, China). All samples were shipped under cold chain management to a central laboratory (certified by the College of American Pathologists), centrifuged, and frozen at -20 °C within 2 h.

### Outcome definitions

Education was divided into completion of a high school education, and lower or beyond high school. Age was categorized into < 45 years and ≥ 45 years [23]. BMI was classified into < 24 kg/m^2^ and ≥ 24 kg/m^2^ [24]. Smoking status was classified as current smokers (past consumption amounted to at least 100 cigarettes and the person was currently smoking), former smokers (quit smoking for more than 6 months), and non-smokers [25]. Alcohol consumption was reported as standard drinks and converted to grams by multiplying by 14. It was considered an abused drink if > 30 g/day for men and > 20 g/day for women [26]. The definition of hypertension was systolic blood pressure ≥ 140 mmHg or diastolic blood pressure ≥ 90 mmHg, and/or self-reported previous diagnosis of hypertension by physicians [26]. Dyslipidemia was defined as TC ≥ 6.22 mmol/L, TG ≥ 2.26 mmol/L, LDL ≥ 4.14 mmol/L, HDL < 1.04 mmol/L, and/or a self-reported previous physician-diagnosed hyperlipidemia as before [27]. Diabetes was defined as FPG level ≥ 7.0 mmol/L, PPG ≥ 11.1 mmol/L, or HbA1c ≥ 6.5% and/or having a self-reported diagnosis of diabetes as the previous study [1, 28].

### Statistical analysis

The baseline characteristics of participants were summarized as mean ± standard deviation or median (interquartile range [IQR]) for continuous variables, and frequencies for categorical variables. Differences between the groups were calculated using the Student’s t-test, the Mann-Whitney U test, and the chi-squared test. Data of essential element concentrations were transformed to natural logarithm for further analysis.

Pearson’s correlation analysis was performed to determine the relationship between the essential element concentrations (natural log-transformed). Pearson’s correlation coefficients were classified as strong (*r* > 0.8), medium (> 0.3 and ≤ 0.8), and weak (*r* ≤ 0.3) [29]. The weak correlations between the essential elements were found using Pearson’s correlation analysis (r: 0.02–0.26, Supplementary Fig. 2). Thus, multivariable logistic regression and linear regression were performed to evaluate the associations of essential element levels with the prevalence of diabetes, FPG, PPG, and HbA1c, considering multiple-element analysis (including all essential elements simultaneously). The essential elements were divided into quartiles, and the lowest quartile was considered as the reference. The dose-response relationships of essential elements with the prevalence of diabetes, FPG, PPG, and HbA1c were detected by the restricted cubic spline (RCS) analysis using a three-knot restricted cubic spline function (with knots at the 10th, 50th, and 90th percentiles). The full model of all the analyses was adjusted for age categories, sex, educational level, smoking status, alcohol consumption, BMI categories, hypertension, and dyslipidemia via directed acyclic graphs (DAGs) (Supplementary Fig. 3). DAGs, also known as causal graph, is an approach based on literature review to determine the minimum sufficient adjustment set in causality and identify confounders [30, 31]. Subgroup analysis was applied to find the potential associations between the essential elements and the prevalence of diabetes among age and sex categories. The covariates were tested without collinearity (all the VIF < 2) (Supplementary Table 1) according to the variance inflation factor (VIF) < 5 [32].

All data were analyzed using IBM SPSS Statistics (version 24) and R (version 4.2.2). A two-tailed *P* value < 0.05 was considered statistically significant.

## Results

### General characteristics of participants with or without diabetes

Table 1 shows the characteristics of 8392 participants including 1066 participants with diabetes and 7326 participants without diabetes. The mean age of diabetes people was 53.42 and 45.52 in no diabetes people. Compared with people without diabetes, the people with diabetes had higher BMI levels, alcohol abuse, and the prevalence of hypertension and dyslipidemia (all *P* < 0.05). In addition, serum Ca, Fe, Cu, and Zn levels were significantly higher in people with diabetes (all *P* < 0.05).

**Table 1:** General characteristics of participants in the study

	Total	Diabetes	No diabetes	*P*
**N**	8392	1066	7326	
**Age, years**	46.53 ± 11.97	53.42 ± 11.22	45.52 ± 11.75	**< 0.001**
**BMI, kg/m** ^**2**^	23.64 ± 3.42	25.07 ± 3.72	23.43 ± 3.32	**< 0.001**
**Men, %**	36.3	40.2	35.8	**0.005**
**FPG, mmol/L**	4.76 ± 1.02	6.02 ± 2.04	4.58 ± 0.57	**< 0.001**
**PPG, mmol/L**	7.89 ± 2.90	13.27 ± 3.84	7.11 ± 1.64	**< 0.001**
**HbA1c, %**	5.68 ± 0.62	6.48 ± 1.15	5.56 ± 0.38	**< 0.001**
**Education, %**				**< 0.001**
< high school	37.5	51.3	35.5	
high school	24.4	24.4	24.4	
> high school	38.1	24.3	40.1	
**Smoking status, %**				**0.035**
No	86.9	84.5	87.3	
Ever	2.9	3.1	2.8	
Current	10.2	12.4	9.9	
**Alcohol abuse, %**	2.8	3.9	2.6	**0.022**
**Hypertension, %**	26.8	46	24	**< 0.001**
**Dyslipidemia, %**	35.3	53.8	32.7	**< 0.001**
**Mg, mmol/L**	0.90(0.86,0.93)	0.89(0.85,0.94)	0.90(0.86,0.93)	0.482
**Ca, mmol/L**	2.38(2.32,2.45)	2.40(2.33,2.47)	2.38(2.31,2.45)	**< 0.001**
**Fe, µmol/L**	15.90(11.80,20.10)	16.80(13.10,20.80)	15.70(11.60,20.00)	**< 0.001**
**Cu, µmol/L**	16.28(14.60,18.04)	16.86(15.12,18.89)	16.20(14.54,17.93)	**< 0.001**
**Zn, µmol/L**	13.99(12.89,15.17)	14.16(13.06,15.25)	13.96(12.87,15.14)	**0.016**

The general characteristics of participants were summarized as mean ± standard deviation or median and interquartile range (IQR) for continuous variables and frequencies for categorical variables (%). Differences between the groups were calculated using the Student’s t-test, the Mann-Whitney U test, and the chi-squared test

BMI: body mass index; FPG: fasting plasma glucose; PPG: 2-h postprandial plasma glucose; HbA1c: glycated hemoglobin; Mg: magnesium; Ca: calcium; Fe: iron; Cu: copper; Zn: zinc

### Associations of serum essential elements and diabetes using multivariable logistic regression and linear regression

**Fig. 1:**
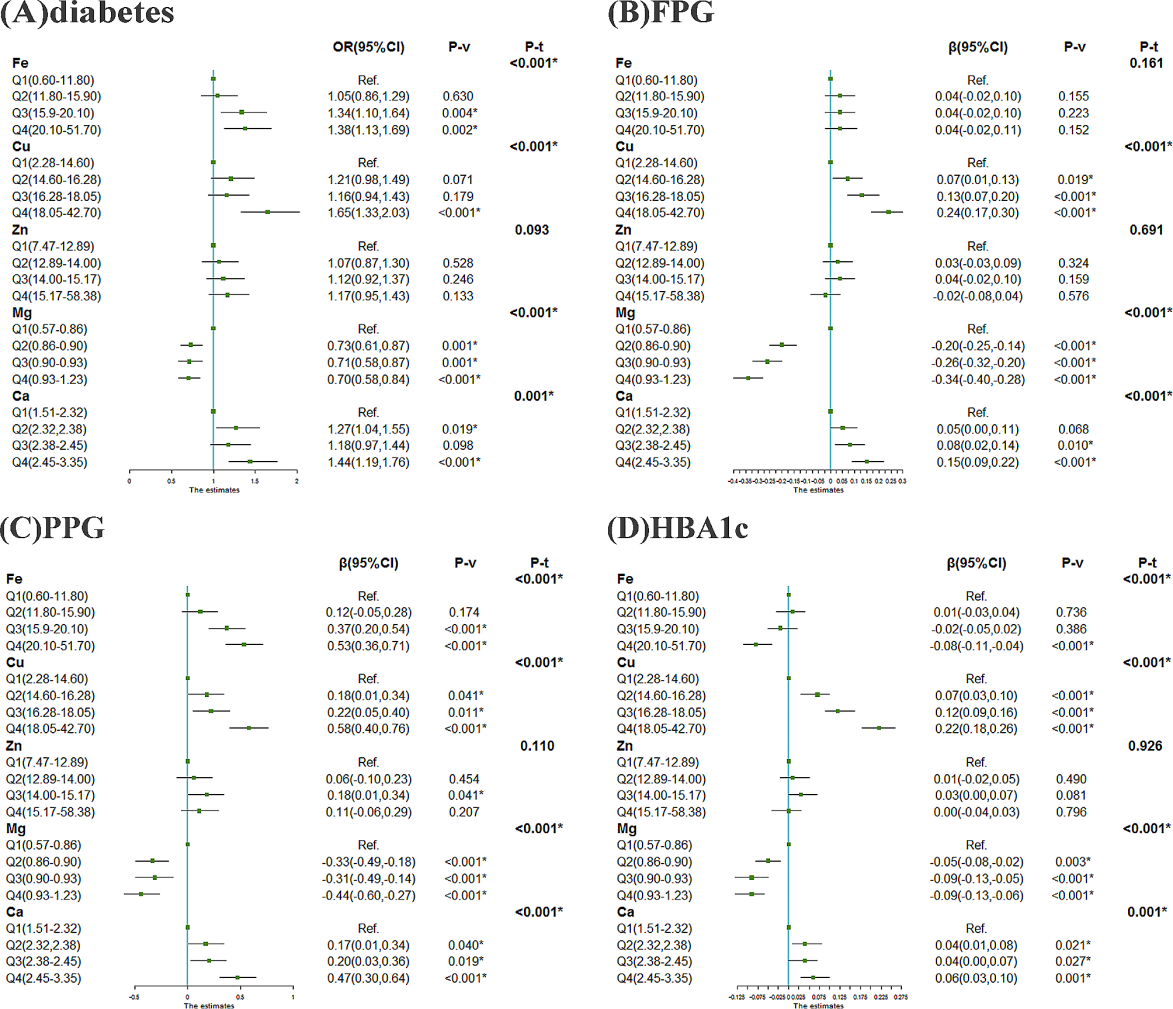
Associations of essential elements with diabetes, FPG, PPG, and HbA1c using multivariable regression (**A**) essential elements and diabetes by logistic regression analysis; (**B**) essential elements and FPG by linear regression analysis; (**C**) essential elements and PPG by linear regression analysis; (**D**) essential elements and HbA1c by linear regression analysis; The essential elements were divided into quartiles, and the lowest quartile was considered as the reference. The full model was adjusted for age categories, sex, educational level, smoking status, alcohol consumption, BMI categories, hypertension, and dyslipidemia FPG, fasting plasma glucose; PPG, 2-h postprandial plasma glucose; HbA1c, glycated hemoglobin; Mg, magnesium; Ca, calcium; Fe, iron; Cu, copper; Zn, zinc; P-v, P for value; P-t, P for trend

Figure 1A shows the associations between serum essential elements and the prevalence of diabetes using multivariable logistic regression. Compared with the lowest quartile, the participants in the highest quartile of Fe, Cu, and Ca possibly had the highest prevalence of diabetes for ORs of 1.38, 1.65, and 1.44, respectively (all *P* < 0.05). However, participants in the highest quartile of Mg had the lowest prevalence of diabetes [0.70(0.58, 0.84), *P* < 0.001]. In addition, to investigate the associations with serum essential elements of FPG, PPG, and HbA1c, multivariable linear regression was used (Fig. 1B-D). We found that the highest quartile of Fe had the highest β [0.53(0.36, 0.71), *P* < 0.001] for PPG, whereas the lowest β [-0.08(-0.11, -0.04), *P* < 0.001] for HbA1c, and the association between Fe and FPG was not found. Compared with the first quartiles, the β value grew as the quartiles of Cu and Ca increased for FPG and PPG, and the β value decreased as the quartiles of Mg increased for FPG and HbA1c (all *P* for trend < 0.001). However, the third quartile of Mg had the highest β for PPG (*P* < 0.001).

**Fig. 2:**
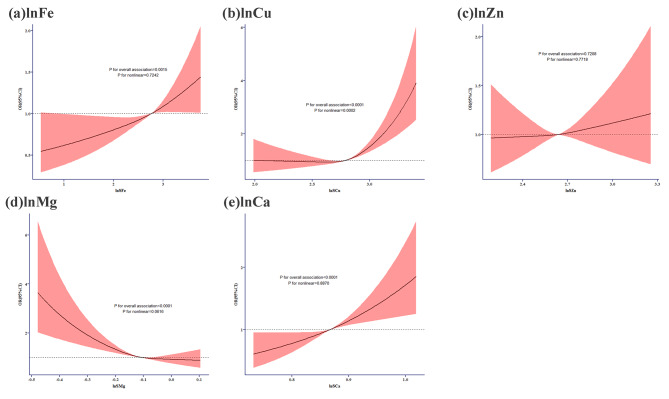
Nonlinear relationships of essential elements in serum with diabetes using RCS analysis (**a**) Fe and diabetes; (**b**) Cu and diabetes; (**c**) Zn and diabetes; (**d**) Mg and diabetes; (**e**) Ca and diabetes; The levels of studied metal elements were ln-transformed to improve normal distribution The full model was adjusted for age categories, sex, educational level, smoking status, alcohol consumption, BMI categories, hypertension, and dyslipidemia Mg, magnesium; Ca, calcium; Fe, iron; Cu, copper; Zn, zinc

### Nonlinear relationships of serum essential elements with diabetes using RCS analysis

Figure 2 represents the dose-response relationships between essential elements and the prevalence of diabetes. It showed that serum Cu displayed an inverse L-shaped relationship with the prevalence of diabetes (*P* for nonlinear < 0.001). However, the associations between the other elements and the prevalence of diabetes were in line (all *P* for nonlinear > 0.05). What’s more, serum Cu and Mg showed a nonlinear relationship with FPG (both *P* for nonlinear < 0.05), serum Fe and Cu displayed an inverse L-shaped relationship with PPG (both *P* for nonlinear < 0.001), serum Fe showed a nonlinear relationship with a sharp decrease following a slight increase with HbA1c (*P* for nonlinear < 0.001) and serum Cu also revealed nonlinear relationship with HbA1c (*P* for nonlinear < 0.05) (Supplementary Fig. 4).

### **Associations of serum essential elements with diabetes using subgroup analysis**

**Fig. 3:**
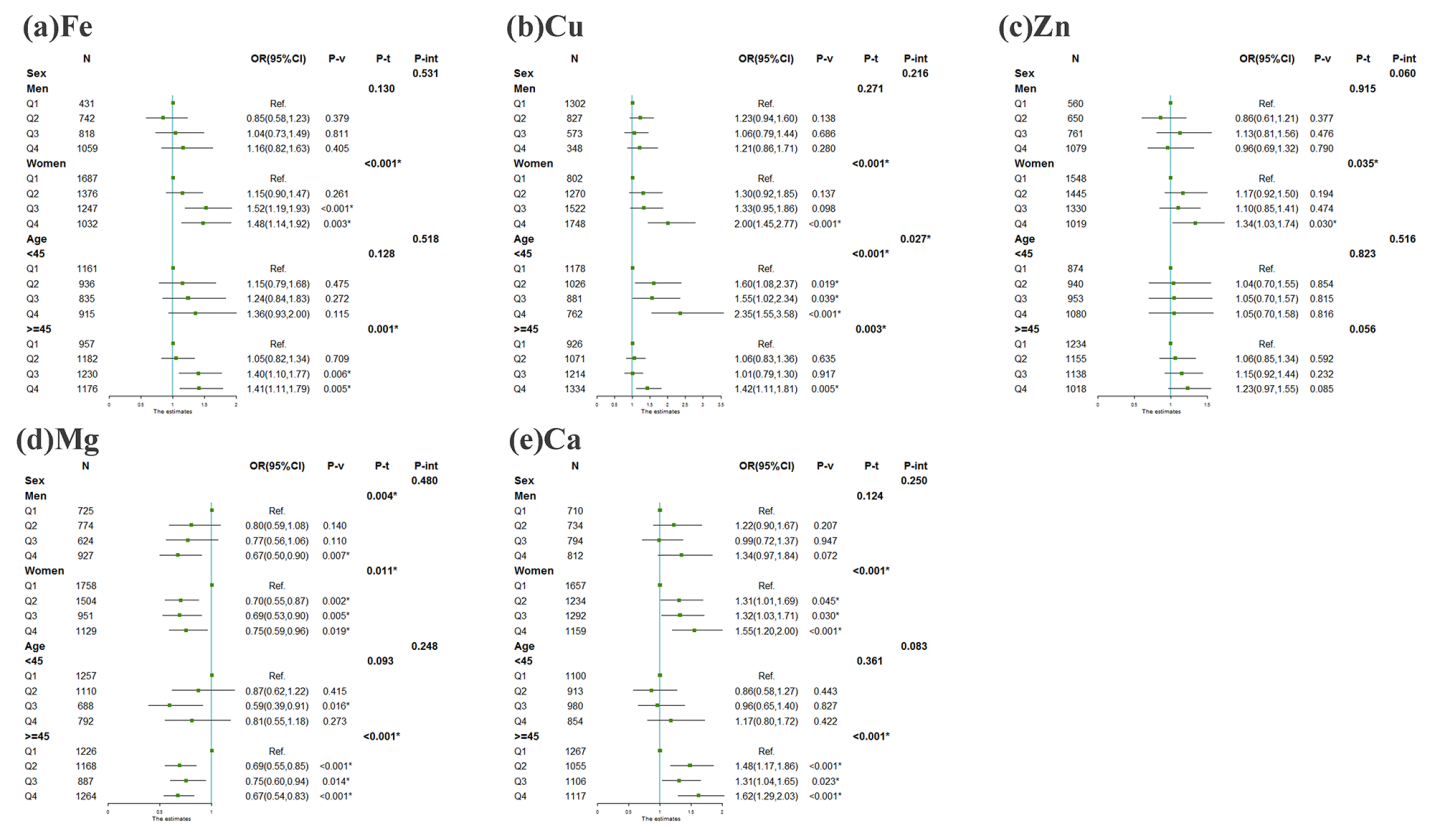
Associations of essential elements in serum with diabetes using subgroup analysis (**a**) Fe and diabetes; (**b**) Cu and diabetes; (**c**) Zn and diabetes; (**d**) Mg and diabetes; (**e**) Ca and diabetes; The essential elements were divided into quartiles, and the lowest quartile was considered as the reference The model was adjusted for age categories, educational level, smoking status, alcohol consumption, BMI categories, hypertension, and dyslipidemia when sex was analyzed in groups. The model was adjusted for sex, educational level, smoking status, alcohol consumption, BMI categories, hypertension, and dyslipidemia when age categories were analyzed in groups Mg, magnesium; Ca, calcium; Fe, iron; Cu, copper; Zn, zinc; P-v, P for value; P-t, P for trend; P-int, P for interaction

Supplementary Table 2 showed that the concentrations of some serum essential elements were significantly different between sex and age groups. Cu was higher in women while the other elements were higher in men (all *P* < 0.001). Serum Fe, Cu, and Mg were higher among the participants in the age group ≥45 years. Figure 3 presented the sex- and age-specific associations between essential elements and the prevalence of diabetes. The significant interactive effect between Cu and age group for the prevalence of diabetes was shown (*P* for interaction = 0.027). In women, the highest quartile of Cu, Zn, and Ca had the highest prevalence of diabetes and the third quartile of Fe had the highest prevalence of diabetes while the third quartile of Mg had the lowest prevalence of diabetes. However, no statistically significant association between serum Fe, Cu, Zn, and Ca and the prevalence of diabetes in men was found. Surprisingly, in the age group < 45 years, a positive association of diabetes with Cu and a negative association with the third quartile of Mg were found. In addition, Supplementary Fig. 5 presented the associations of serum Cu with FPG, PPG, and HbA1c in subgroup analyses. We found the interactive effect between Cu and age group for PPG (*P* for interaction = 0.002). What’s more, the β value grew as the quartiles of Cu increased for FPG, PPG, and HbA1c (all *P* for trend < 0.05).

## Discussion

This study investigated the associations of five essential elements including Fe, Cu, Zn, Mg, and Ca with the prevalence of diabetes as well as FPG, PPG, and HbA1c in the community of China. It was found that serum Cu was positively associated with FPG, PPG, and HbA1c while serum Mg was significantly inversely correlated with FPG, PPG, HbA1c, and diabetes. Moreover, the non-linear relationships of Cu and diabetes, FPG, PPG and HbA1c, Mg and FPG, Fe and PPG, and Fe and HbA1c were found. Furthermore, stronger positive associations of Cu with diabetes and PPG were found in younger women.

Magnesium (Mg^2+^), one of the most abundant essential elements in the body, is a cofactor of more than 300 enzymes involved in a variety of metabolic processes, including energy production, blood glucose regulation, and blood pressure control [33, 34]. One previous study demonstrated that the serum Mg was inversely associated with diabetes [11]. Other studies also found a negative relationship of plasma Mg with FPG which was consistent with our research [12, 35]. Interestingly, in our study, the negative association between serum Mg and diabetes was particularly pronounced in women. This discovery was consistent with the result of another prospective study [12, 36], suggesting a sex-dependent effect of Mg on diabetes.

The mechanism by which hypomagnesemia increases the risk of diabetes is not fully established, but there are several possible mechanisms. In an animal experiment, Mg treatment can suppress the expression of gluconeogenic genes, such as the phosphoenol pyruvate carboxykinase (PEPCK) gene, and then reduce blood glucose [37]. Furthermore, the lack of Mg^2+^ will decrease the activity of insulin receptor tyrosine kinase, then obstruct the intracellular signaling pathway, reduce insulin sensitivity, and eventually lead to diabetes [34, 38]. Meanwhile, the disturbance of the pathway may also reduce glucose uptake in target tissues stimulated by glucose transporter type 4 (GLUT4). And the gene expression of GLUT4 can be increased by Mg^2+^ [39].

Cu, the third most abundant essential metal in our bodies, is of great importance to various enzymatic reactions, participating in redox reactions [40]. Although Cu is an important component of antioxidant defense enzymes (such as Cu/Zn SOD), more and more studies have indicated that Cu has a role in promoting oxidative stress [41]. For example, a study showed that high Cu intake induced oxidative stress in diabetic rats [42]. Zheng, Yinnan, et al. found that plasma Cu was positively associated with glucose levels among pregnant women [43]. A prospective cohort study in Japanese adults suggested that dietary Cu intake was positively associated with type 2 diabetes (T2D) risk [44]. The current study indicated that serum Cu was significantly higher in people with T2D which was consistence with the previous studies. However, another study in the Chinese population suggested that dietary intake of Cu was not significantly associated with T2D risk [45]. There are several possible reasons for the above difference. Firstly, through the small intestine, Cu is absorbed into the liver from the diet, and 80% is excreted through the biliary tract [46]. Therefore, dietary intake of Cu does not accurately reflect the status and content of Cu in the body. Secondly, our current study found the interactive effect between Cu and age group for diabetes and PPG. Therefore, the age composition of the population is an easily overlooked factor affecting the relationship of Cu with diabetes.

It is reported that Ca plays an important role in insulin resistance and secretion [15]. On one hand, insulin secretion is a Ca-dependent process that was triggered by extracellular Ca to enter pancreatic β cells through voltage-gated calcium channels [47]. On the other hand, increased Ca levels can reduce insulin sensitivity by decreasing the expression of glucose transporters (GLUT4) in myotubes and adipocytes. Therefore, the uptake of glucose was decreased and the plasma glucose increased eventually [15]. In a previous study, a case-control study in the Chinese population indicated that serum Ca was positively and nonlinearly associated with T2D risk [48]. Furthermore, a prospective cohort study also found that increased serum Ca was at greater risk of T2D by following up for 8.8 years [49]. In the current study, elevated serum Ca was found associated with an increased prevalence of diabetes, and a higher level of FPG, PPG, and HbA1c which was consistent with the previous studies. However, a prospective cohort in the Korean population aged 40–69 years showed no significant relationship between serum Ca and T2D [50]. And the possible reasons may be differences in sample size and age composition.

Fe plays a crucial role in oxygen transport and metabolic regulation [6]. Plenty of studies have shown that Fe overload is one of the risk factors for diabetes [51–53]. On one hand, Fe^2+^ can promote ROS production through the Fenton reaction, and eventually lead to apoptosis of pancreatic β cells which are sensitive to oxidative stress [6]. On the other hand, Fe overload can reduce insulin sensitivity in insulin-sensitive tissues such as liver, muscle, and fat, and disrupt glucose metabolism, ultimately leading to diabetes [53]. Several cohort studies suggested that plasma ferritin concentration was positively associated with diabetes [54, 55]. We further revealed a positive correlation between serum Fe and diabetes in women but not in men. These results suggested that the correlation between diabetes and Fe status is sex-specific and women have a higher prevalence of diabetes than men.

What’s more, we found a negative association between serum Fe and HbA1c, which was consistent with the results in two Mendelian randomization studies [56, 57]. Studies showed that high levels of HbA1c were related to iron deficiency anemia [58, 59]. The possible reasons may be as follows: firstly, the concentration of HbA1c elevated for the increased age of erythrocyte which was positively associated with iron deficiency [60, 61]. Secondly, HbA1c is formed by the glycation of the valine at the hemoglobin-chain terminal [59], which will be glycosylated more easily in relative iron deficiency [58].

In the current study, the serum Fe, Cu, Zn, and Ca were positively associated with the prevalence of diabetes in women, but not in men. Sex-specific associations of metals with FPG or HbA1c have been reported previous studies [12, 16]. These sex differences may be due to the different cumulative effects of metal elements across different periods of growth in men and women [62]. Our findings of significant correlations between diabetes and serum Fe, Mg, and Ca among participants aged ≥45 may support this hypothesis. Additionally, the endogenous sex hormones may also play an important role in the sex difference of diabetes. It is reported that high levels of testosterone are positively associated with diabetes in women but negatively in men [63]. What’s more, genetic factors may also be another possibility. For example, the T2D-related genes influence more on T2D development in men than women [64].

There remain several limitations in the current study. Firstly, the causal relationship between essential elements and diabetes risk cannot be determined for a cross-sectional study, and more prospective cohort studies are needed for further research. Secondly, we only detected five essential metals that are frequently tested clinically, and more elements need to be measured. Thirdly, metal elements play a role as ions and in combination with proteins in the body, and the measurement of serum element concentrations may cause deviations. Finally, although confounders were screened using DAGs, the adjusted confounders are not sufficient.

## Conclusions

The current study focused on the association of essential elements with diabetes diagnosed according to an OGTT and HbA1c in a sex- and age-specific manner. This study showed that people with higher levels of serum Fe, Cu, and Ca and lower levels of serum Mg had a greater prevalence of diabetes, especially women. Stronger positive associations of Cu with diabetes and PPG were found in younger women. These findings may lead to more appropriate approaches to essential elements supplementation in people with diabetes of different ages and sexes. However, more prospective cohort and experimental studies are needed to probe the possible mechanism of sex- and age-specific associations between serum essential elements and diabetes.

### Electronic supplementary material

Below is the link to the electronic supplementary material.

Supplementary Material 1
**Supplementary Fig. 1.** Flow chart of the study population selected
**Supplementary Fig. 2.** The Correlations between different serum essential elements
The correlations between the essential elements were found using Pearson’s correlation analysis. The higher the levels of correlation coefficients, the darker the color represents
The levels of studied metal elements were ln-transformed to improve normal distribution
**Supplementary Fig. 3.** Directed Acyclic Graphs for variable screening
Red circles represented confounding factors (age categories, sex, educational level, smoking status, alcohol consumption, BMI categories, hypertension, and dyslipidemia), green circle represented exposure (serum metal elements); blue circle represented outcome (diabetes)
**Supplementary Fig. 4.** Nonlinear relationships of serum essential elements with FPG, PPG, and HbA1c using RCS analysis
(A) essential elements and FPG; (B) essential elements and PPG; (C) essential elements and HbA1c;
The levels of studied metal elements were ln-transformed to improve normal distribution
The full model was adjusted for age categories, sex, educational level, smoking status, alcohol consumption, BMI categories, hypertension, and dyslipidemia
FPG, fasting plasma glucose; PPG, 2-h postprandial plasma glucose; HbA1c, glycated hemoglobin; Mg, magnesium; Ca, calcium; Fe, iron; Cu, copper; Zn, zinc
**Supplementary Fig. 5.** Associations of serum Cu with FPG, PPG, and HbA1c using subgroup analysis
(A) Cu and FPG; (B) Cu and PPG; (C) Cu and HbA1c;
The serum Cu was divided into quartiles, and the lowest quartile was considered as the reference
The model was adjusted for age categories, educational level, smoking status, alcohol consumption, BMI categories, hypertension, and dyslipidemia when sex was analyzed in groups. The model was adjusted for sex, educational level, smoking status, alcohol consumption, BMI categories, hypertension, and dyslipidemia when age categories were analyzed in groups
FPG, fasting plasma glucose; PPG, 2-h postprandial plasma glucose; HbA1c, glycated hemoglobin; Mg, magnesium; Ca, calcium; Fe, iron; Cu, copper; Zn, zinc; P-v, P for value; P-t, P for trend; P-int, P for interaction
**Supplementary Table 1.** The collinearity diagnosis of the covariates
**Supplementary Table 2** Mean rank of serum elements among different groups

## Data Availability

The data and material supporting the findings of the study are available from the corresponding authors upon reasonable request.

## Abbreviations

OGTT: oral glucose tolerance test
HbA1c: glycated hemoglobin
Fe: iron
Cu: copper
Zn: zinc
Mg: magnesium
Ca: calcium
FPG: fasting plasma glucose
***PPG***: 2-h postprandial plasma glucose
***RCS***: restricted cubic spline
DAGs: directed acyclic graphs
BMI: body mass index
TC: total cholesterol
TG: triglyceride
HDL: high-density lipoprotein
LDL: low-density lipoprotein
PEPCK: phosphoenol pyruvate carboxykinase
GLUT4: glucose transporter type 4
T2D: type 2 diabetes
