# Supplementary material for: Sex- and age-specific associations of serum essential elements with diabetes among the Chinese adults: a community-based cross-sectional study
Source: Nutr Metab (Lond). 2024 Jul 9;21:44. doi: 10.1186/s12986-024-00801-3 (PMC11232217; doi:10.1186/s12986-024-00801-3)

**Supplementary Table 1.** The collinearity diagnosis of the covariates

| Characteristic | VIF |
| --- | --- |
| Age | 1.383 |
| Sex | 1.657 |
| Education level | 1.321 |
| BMI categories | 1.110 |
| Smoking status | 1.356 |
| Abused drink | 1.075 |
| Hypertension | 1.129 |
| Dyslipidemia | 1.137 |
| lnMg | 1.076 |
| lnCa | 1.123 |
| lnFe | 1.110 |
| lnCu | 1.203 |
| lnZn | 1.161 |

Data are presented as variance infation factor (VIF).

**Supplementary Table 2.** Mean rank of serum elements among different groups

|  | N(%) | Fe | Cu | Zn | Mg | Ca |
| --- | --- | --- | --- | --- | --- | --- |
| **Sex** |  |  |  |  |  |  |
| Men | 3050(36.34) | 4971.60 | 3051.92 | 4811.67 | 4618.59 | 4492.52 |
| Women | 5342(63.66) | 3753.96 | 4850.00 | 3845.27 | 3955.51 | 4027.49 |
| ***P*** |  | <0.001 | <0.001 | <0.001 | <0.001 | <0.001 |
| **Age group** |  |  |  |  |  |  |
| <45 | 3847(45.84) | 3976.83 | 3806.03 | 4397.70 | 3947.07 | 4152.52 |
| >=45 | 4545(54.16) | 4382.44 | 4527.01 | 4026.20 | 4407.62 | 4233.72 |
| ***P*** |  | <0.001 | <0.001 | <0.001 | <0.001 | 0.13 |

**Supplementary Figure 1.** Flow chart of the study population selected.


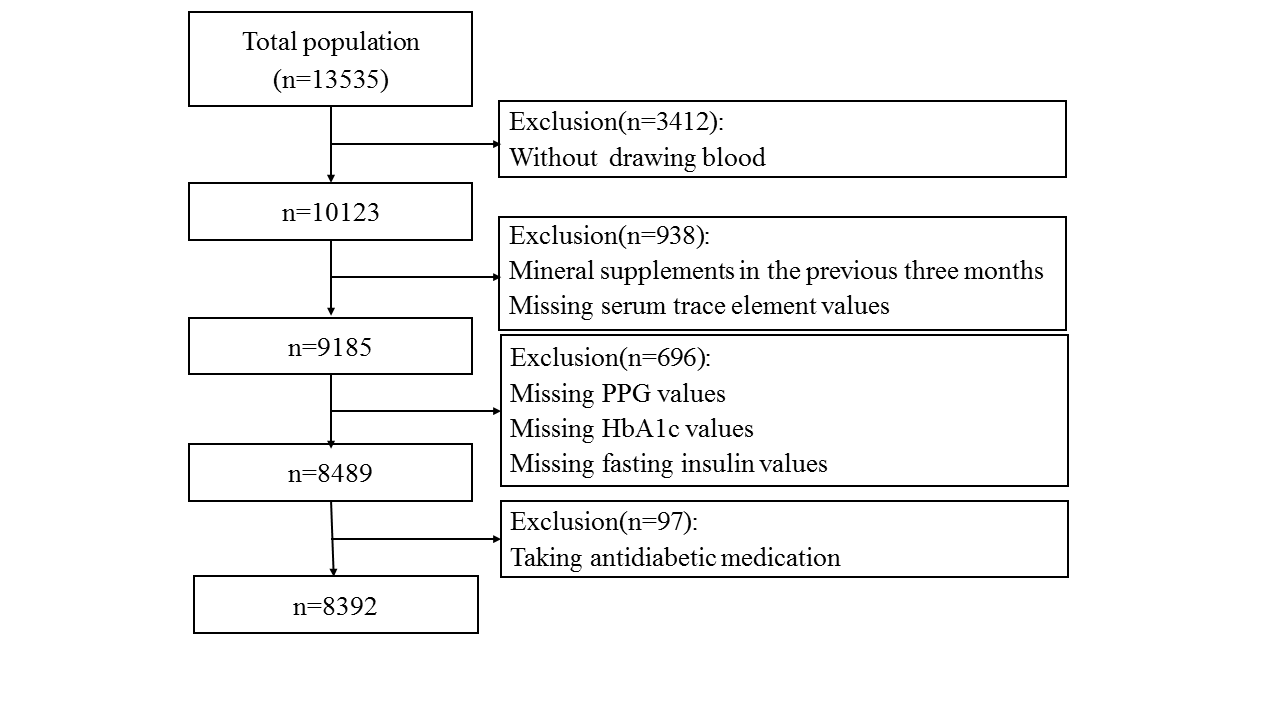


**Supplementary Figure 2.** The Correlations between different serum essential elements


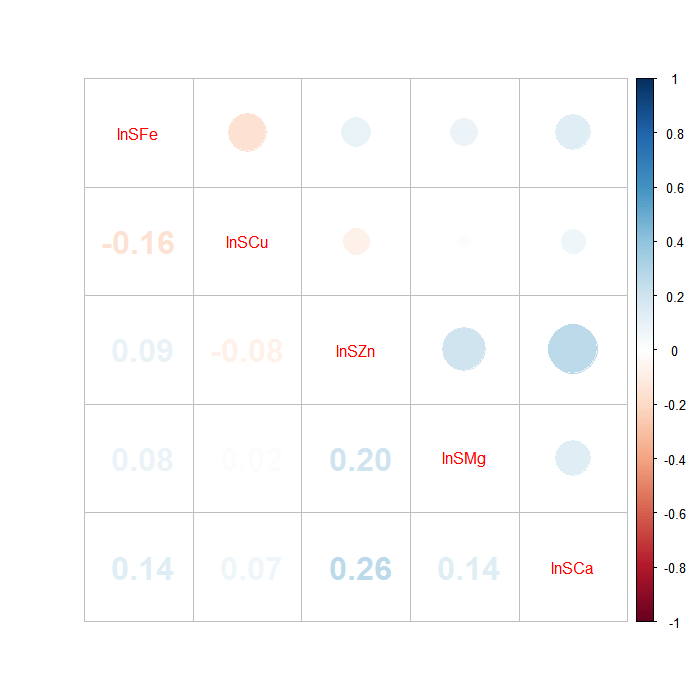


**Supplementary Figure 3.** Directed Acyclic Graphs for variable screening

**
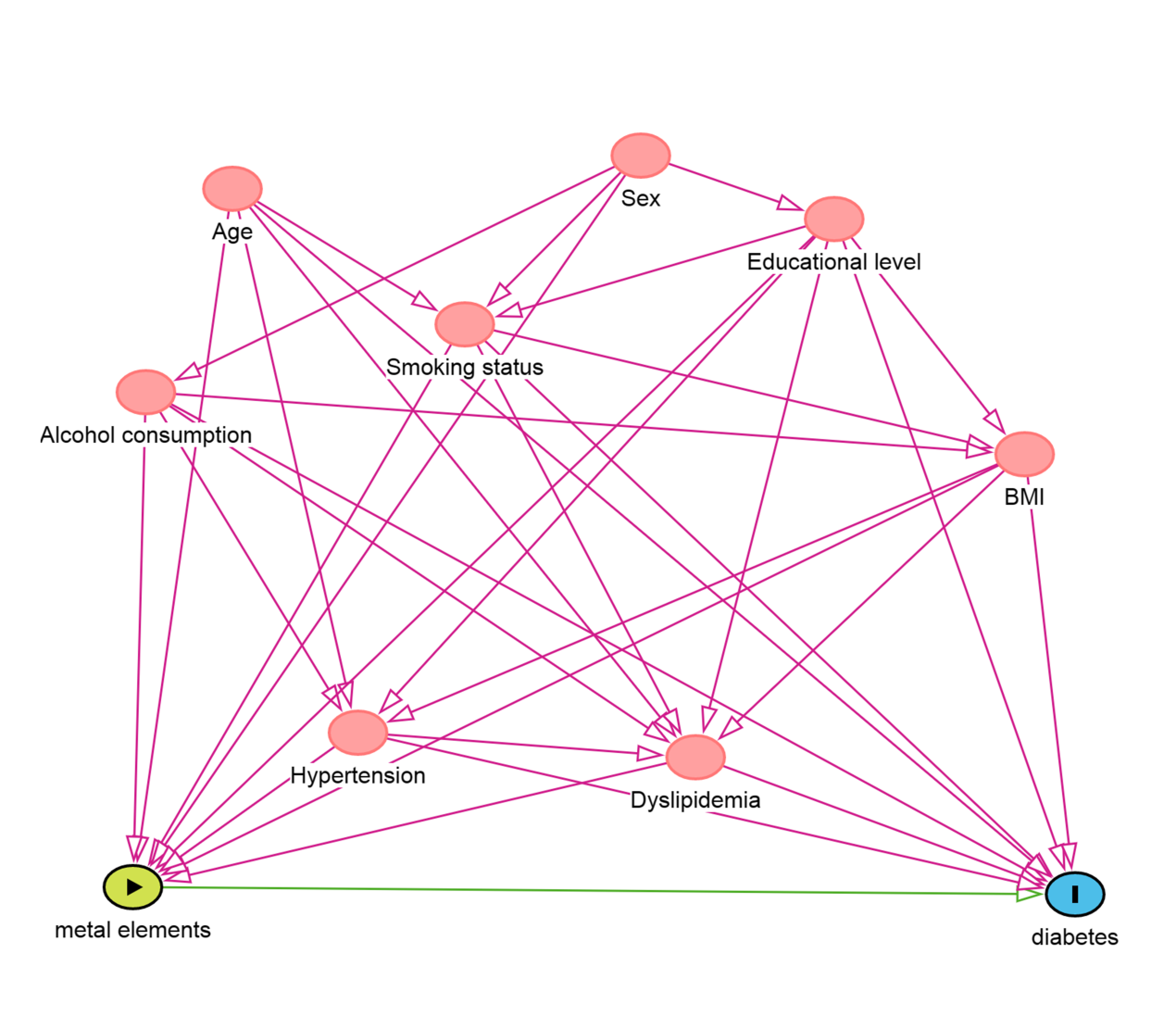
**

**Supplementary Figure 4.** Nonlinear relationships of serum essential elements with FPG, PPG, and HbA1c using RCS analysis


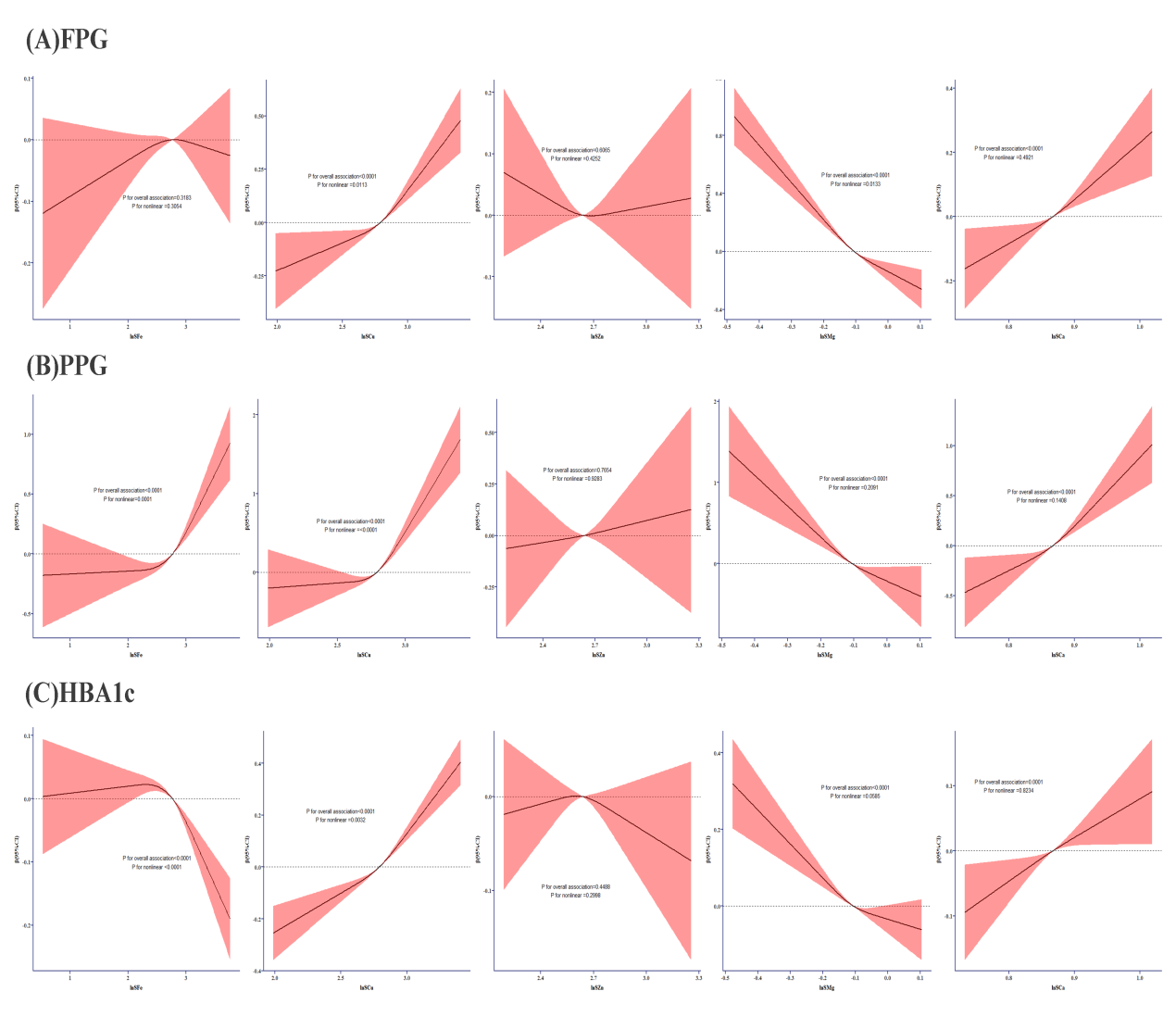


**Supplementary Figure 5.** Associations of serum Cu with FPG, PPG, and HbA1c using subgroup analysis


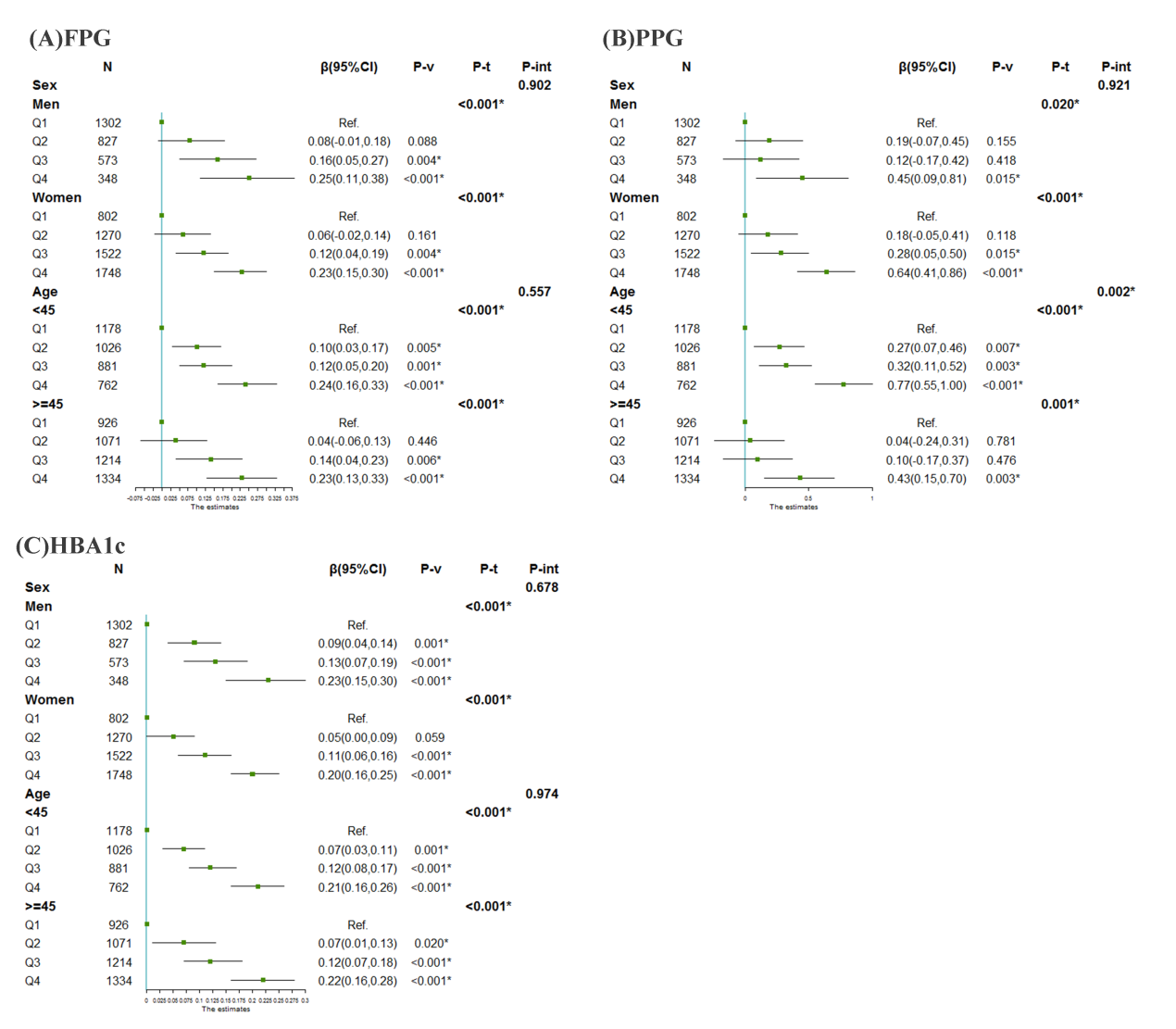

Supplement: Supplementary file 1 — Supplementary Material 1 Supplementary Fig. 1. Flow chart of the study population selected Supplementary Fig. 2. The Correlations between different serum essential elements The correlations between the essential elements were found using Pearson’s correlation analysis. The higher the levels of correlation coefficients, the darker the color represents The levels of studied metal elements were ln-transformed to improve normal distribution Supplementary Fig. 3. Directed Acyclic Graphs for variable screening Red circles represented confounding factors (age categories, sex, educational level, smoking status, alcohol consumption, BMI categories, hypertension, and dyslipidemia), green circle represented exposure (serum metal elements); blue circle represented outcome (diabetes) Supplementary Fig. 4. Nonlinear relationships of serum essential elements with FPG, PPG, and HbA1c using RCS analysis (A) essential elements and FPG; (B) essential elements and PPG; (C) essential elements and HbA1c; The levels of studied metal elements were ln-transformed to improve normal distribution The full model was adjusted for age categories, sex, educational level, smoking status, alcohol consumption, BMI categories, hypertension, and dyslipidemia FPG, fasting plasma glucose; PPG, 2-h postprandial plasma glucose; HbA1c, glycated hemoglobin; Mg, magnesium; Ca, calcium; Fe, iron; Cu, copper; Zn, zinc Supplementary Fig. 5. Associations of serum Cu with FPG, PPG, and HbA1c using subgroup analysis (A) Cu and FPG; (B) Cu and PPG; (C) Cu and HbA1c; The serum Cu was divided into quartiles, and the lowest quartile was considered as the reference The model was adjusted for age categories, educational level, smoking status, alcohol consumption, BMI categories, hypertension, and dyslipidemia when sex was analyzed in groups. The model was adjusted for sex, educational level, smoking status, alcohol consumption, BMI categories, hypertension, and dyslipidemia when age categories were analyzed in groups FPG [file 12986_2024_801_MOESM1_ESM.docx]
